# Detection of postural control in early Parkinson’s disease: Clinical testing vs. modulation of center of pressure

**DOI:** 10.1371/journal.pone.0245353

**Published:** 2021-01-12

**Authors:** Anna Kamieniarz, Justyna Michalska, Wojciech Marszałek, Magdalena Stania, Kajetan J. Słomka, Agnieszka Gorzkowska, Grzegorz Juras, Michael S. Okun, Evangelos A. Christou

**Affiliations:** 1 Institute of Sport Sciences, Academy of Physical Education, Katowice, Poland; 2 Department of Neurorehabilitation, Faculty of Medical Sciences in Katowice, Medical University of Silesia, Katowice, Poland; 3 Department of Neurology, Norman Fixel Institute for Neurological Diseases, University of Florida, Gainesville, FL, United States of America; 4 Department of Applied Physiology and Kinesiology, University of Florida, Gainesville, FL, United States of America; Emory University, UNITED STATES

## Abstract

**Introduction:**

Little is known about the early stage balance changes in PD. Many clinicians assume that there are no postural issues in early PD because of failure to identify them on bedside and clinical testing. Here, we quantify balance changes in early and moderate stage PD and compared these values to healthy controls (HC) using clinical assessments of balance and posturography.

**Methods:**

We compared 15 HC with 15 early PD (PD-II; Hoehn and Yahr stage II) and 15 moderate PD (PD-III; H&Y stage III). Participants performed various clinical tests of balance and a standing postural task on a force platform. We quantified the spatiotemporal parameters of the center of pressure (COP), the sample entropy and power spectral density (PSD) of the COP.

**Results:**

The PSD of the COP differentiated PD-II from HC from 0–0.5 Hz and PD-II from PD-III from 0.5–1 Hz. Specifically, PD-II and PD-III manifested greater power than HC from 0–0.5 Hz, whereas PD-III exhibited greater power than PD-II and HC from 0.5–1.0 Hz (p<0.05). However, there were no significant differences between PD-II and HC in all clinical tests and in spatiotemporal parameters of the COP (p>0.05). Although the sample entropy was significantly lower in the PD groups (p<0.05), entropy failed to differentiate PD-II from PD-III.

**Conclusion:**

The low-frequency modulation of the COP in this small cohort differentiated early PD from HC and from moderate PD. Clinicians should be aware that there are early balance deficits in PD. A larger sample size is needed to confirm these findings.

## Introduction

Falling, is a major threat to the safety and well-being of patients with Parkinson’s disease (PD). Indeed, PD patients have a higher risk for falls and this risk is contributed to largely by impairments in postural control and balance [1]. Currently, however, the loss of postural control and balance at the early stages of the disease remain poorly understood. Clinical tests of balance appear to be insensitive in identifying early changes in PD balance. For example, the only available study [2] that compared early stage PD (Hoehn and Yahr scale of 1–2; H&Y) to healthy controls found no differences in the performance of a common clinical test that indirectly includes some elements of balance (“Timed Up and Go Test”). In contrast, early PD patients exhibited greater center of pressure (COP) excursions than healthy controls (HC) on a force platform (posturography) [3]. Although this study suggested that measures of postural control may be required to detect balance impairments in early stage PD, it was limited to only nine patients. Thus, an important, but unresolved question in the literature is the following: “Can we identify balance changes in early stage PD and differentiate them from healthy controls?” Here, we quantified changes in balance and postural control for PD at early (II H&Y) and moderate (III H&Y) stages of the disease relative to healthy controls. We used clinical measures of balance as well as spatiotemporal and frequency measures of postural control.

Changes in postural control have been reported in moderate stages of PD (III H&Y). For example, Ferrazzoli et al. [4] showed that PD individuals exhibited more COP sway as compared with HC. Moreover, Barbieri et al. [5] showed that moderate stage PD exhibited higher postural control asymmetry than mild PD. However, in early stages PD (I and II H&Y), the ability to quantify changes in postural control using clinical measures of balance has been largely disappointing. Salarian et al. [2] showed that neither a TUG test or an instrumented version of the same test was significantly different between early PD and HC. The only evidence we could find was Chastan et al. [3] showing that the area of the COP excursions was greater in early stage PD than in HC. Supporting, but indirect evidence can be gleaned from studies examining the effect of levodopa on postural control. Beuter et al. [6] showed that COP excursions (mean sway, sway area) decreased in response to levodopa treatment in early PD. Thus, it appears that early stage PD patients have postural control deficits but these conclusions are drawn by a few studies that used basic measures of postural control.

There have been significant technical advancements in instrumentation and analytical methods for measuring postural control however [7], the single study in the literature used only the spatiotemporal quantitative measures of COP in 9 patients [3]. The problem with this approach is that COP area can be biased by random excursions. Another problem with these basic posturographic measures is that they can inadvertently capture the amplitude of the COP displacement without enlightening COP control. A better understanding of postural control will require more advanced analytical methods, which will quantify the structure of the COP [8,9].

Spectral analysis of the COP has revealed that the frequency of HC and patients with neurodegenerative diseases is different. Specifically, HC have a dominant sway from 0.1–0.5 Hz [10–12], whereas in moderate PD the dominant frequency manifests at 0–2 Hz and 3–7 Hz [13]. To date most posturography studies in PD focus on the basic spatiotemporal measures (see review by Kamieniarz et al. [14]).

We concluded that there is a paucity of information on changes in postural control and balance in early PD cases. The limitations of the analytical methods used to quantify postural control and balance have contributed to a potential false sense among clinicians that early PD may not have postural control issues. We therefore hypothesized that early PD cases would reveal changes in balance and postural control for PD but that advanced analytical methods using COP measurements would be required to show them.

## Methods

### Participants

The participants in this study were 15 adults with early PD (classified as stage 2 of the Hoehn and Yahr Scale) [15], 15 with moderate PD (classified as stage 3 of the Hoehn and Yahr Scale) and 15 age-matched healthy controls. The participants were recruited from the Department of Neurology, University Clinical Center Medical University of Silesia in Katowice, Poland during the January 2018 to June 2018 study period. The clinical diagnosis of PD was performed by a highly qualified Neurologist with long term experience in PD. Participants were excluded if they had scores <24 in the Mini Mental State Examination [16], had current musculoskeletal injuries, or exhibited vestibular disorders. PD patients were tested during the “ON period” of their usual antiparkinsonian medication (at least one hour after they took their medication) and none of the patients exhibited any dyskinesia or dystonia signs during testing. All subjects provided informed written consent prior to participation in the study. The study was approved by the ethics committee of the Academy of Physical Education in Katowice, Poland (No.7/2013/26.06.2013). The demographic and clinical characteristics of the participants are presented in Table 1.

**Table 1:** The demographic and clinical characteristics of the participants.

	Early PD (PD-II)	Moderate PD (PD-III)	Healthy control (HC)
N	15	15	15
Age [years]	61.9±8.6	70.7±4.5	63.5±4.3
Body mass [kg]	75.7±9.0	75.1±15.0	70.2±7.9
Body height [cm]	170.4±6.4	168.6±5.4	167.3±4.3
UPDRS—III [pts]	10.5±3.4	28.9±5.4	-
H&Y stage	II	III	-
MMSE [pts]	28.1 ± 1.6	27.5 ± 2.5	29.0 ± 1.6

UPDRS—Unified Parkinson’s Disease Rating Scale, H&Y–Hoehn and Yahr Scale, MMSE—Mini Mental State Examination.

### Procedures

Participants performed all procedures in a single session. First, they performed the clinical assessments related to PD followed by assessments of balance using posturography.

#### Clinical assessments

1) UPDRS part III–to quantify the motor impairments related to PD [17]; 2) Berg Balance Scale (BBS)–to assess static balance and fall risk [18]; 3) Timed Up and Go test (TUG)–to measure balance, walking ability, and fall risk [19]; 4) Tinetti test–to assess balance, gait, and fall risk [20]; 5) Functional Reach Test (FRT)–to evaluate dynamic balance, limits of stability, and fall risk [21].

#### Posturography

Participants stood barefoot on a force plate (AMTI Accugait), with the feet placed apart (shoulder width) and the arms hanging relaxed alongside the body. COP was recorded in two experimental conditions: eyes open (OE) and eyes closed (CE). The COP trajectories were recorded in both anterior-posterior (AP) and mediolateral (ML) directions. Each participant performed each condition three times. Trial duration was 30 s. During the eyes open trial participants were instructed to stand still and look straight ahead at a fixed visual reference mark that was located 1.5 m in front of them.

### Data analysis

Center of pressure (COP) displacement time series data were collected at 100 Hz and were processed offline using the Matlab software (Mathworks Inc., Natick, MA, USA) with a 7 Hz, fourth-order, low-pass Butterworth filter. The following posturographic outcomes were extracted:

#### Spatio-temporal measures of COP

1) range of COP (raCOP) in the AP and ML directions indicating the maximum excursion of the COP in cm for each direction; 2) velocity of COP (vCOP) in the AP and ML directions indicating the velocity of the COP sway calculated by dividing the total length of the COP trajectory (in cm) by the recording time length (in seconds); 3) root mean square of COP (rmsCOP) in the AP and ML directions indicating the displacements of the COP around the mean COP (variability of the COP excursion in cm).

#### Sample entropy measures of COP

In this study we used the sample entropy algorithm and the criterion proposed by Richman and Moorman’s [22] and Lake et al. [23] to select the input parameters of the algorithm. Hence, the sample entropy is the negative logarithm of the probability that a dataset of length N, having repeated itself for m samples within a tolerance r, will also repeat itself for m + 1 samples, without allowing self-matches. The parameters m, and r must be fixed for each calculation, m is the length of sequences to be compared, and r is the tolerance for accepting matches. In our study, we used the recommended values of input parameters: m = 3, and r = 0.2 [23,24].

The sample entropy is defined by the following formula:

$$SampEn=-Ln\frac{A^{m}(r)}{B^{m}(r)}$$

Where Ln is the negative logarithm, A is the total number of forward matches of length m + 1, and B is the total number of templates matches of length m.

#### COP oscillations

Similar to Watanabe et al. [12], we quantified COP oscillations using a Fourier analysis and the resulting power spectrum density (PSD). For the Fourier analysis, we detrended and low-pass filtered the anteroposterior and mediolateral COP displacements at 12 Hz (a fourth-order zero phase lag Butterworth filter). The window size was set at 15 s, which gave us a 0.066 Hz resolution based on the length of our time series (30 s). Given that about 80% of the power of the COP time series was within 0–1 Hz for all groups, we divided the PSD of the COP into 2 frequency bins (0–0.5, 0.5–1.0 Hz). The dependent variable was the absolute peak power in each frequency bin.

### Statistical analysis

One-way ANOVA was used to detect significant differences in clinical tests between PD groups and healthy subjects. A mixed-model two-way ANOVA (3 groups × 2 vision) was used to examine raCOP, vCOP, rmsCOP and sample entropy for each direction. We used a mixed-model three-way ANOVA (3 groups × 2 vision × 2 frequency bands) to compare the PSD from the groups across two vision and two frequency bands (0–0.5 and 0.5–1 Hz) for each direction. The alpha level for all statistical test was set at 0.05.

## Results

### Clinical tests

**Fig 1:**
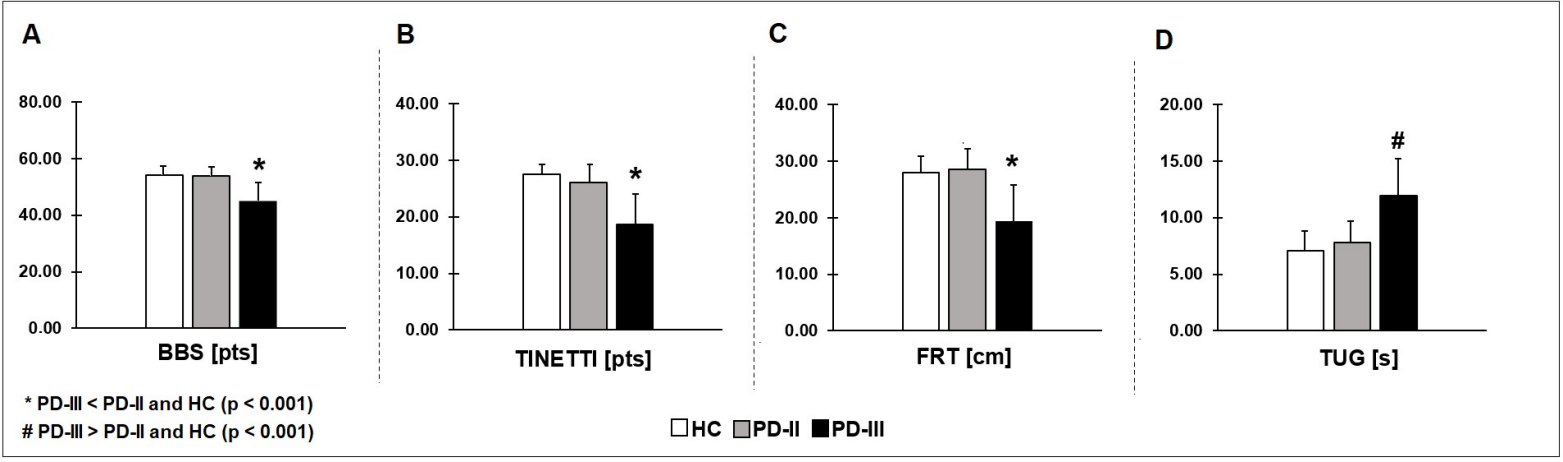
Four different clinical tests of balance among HC, early PD (PD-II), and moderate PD (PD-III). Moderate PD participants exhibited impaired BBS (A), TINETTI (B), FRT (C), and TUG (D) scores relative to HC and early PD. This finding indicates that although clinical tests of balance captured the balance impairments in moderate PD, they were insensitive in identifying balance impairments in early PD relative to HC.

We compared balance among HC, PD-II, and PD-III using four common clinical tests of balance. PD-III participants exhibited impaired BBS (Fig 1A), TINETTI (Fig 1B), FRT (Fig 1C), and TUG (Fig 1D) scores relative to HC and PD-II (Table 2). There were no significant differences between PD-II and HC (P > 0.05). This finding indicates that clinical tests of balance captured the balance impairments only for PD-III relative to HC and PD-II. However, clinical tests of balance were insensitive in detecting balance impairments in PD-II relative to HC.

**Table 2:** Intergroup comparison between three groups HC, PD-II, and PD-III.

	HC	PD-II	PD-III	PD-II vs. HC	PD-III vs. HC	PD-II vs. PD-III
Variables	Mean (SD)	Mean (SD)	Mean (SD)	P-value	P-value	P-value
BBS (pts)	53.67 (2.09)	53.13 (5.67)	45.93 (6.43)	P > 0.05	P < 0.001*	0.001*
TUG (s)	7.15 (1.90)	8.18 (2.09)	11.79 (5.00)	P > 0.05	0.001*	0.014*
FRT (cm)	26.87 (5.42)	28.80 (5.83)	19.66 (7.09)	P > 0.05	0.008*	P < 0.001*
Tinneti (pts)	27.40 (0.74)	25.47 (4.15)	18.40 (6.48)	P > 0.05	P < 0.001*	P < 0.001*
raCOP AP (cm)	2.65 (1.15)	2.67 (0.88)	3.45 (1.23)	P > 0.05	0.019*	0.023*
raCOP ML (cm)	1.72 (0.63)	1.61 (0.68)	2.56 (1.48)	P > 0.05	0.007*	0.002*
rmsCOP AP (cm)	0.52 (0.24)	0.54 (0.18)	0.69 (0.28)	P > 0.05	0.026*	0.048*
rmsCOP ML (cm)	0.33 (0.11)	0.32 (0.14)	0.51 (0.28)	P > 0.05	0.002*	0.001*
vCOP AP (cm/s)	1.04 (0.41)	0.88 (0.31)	1.45 (0.83)	P > 0.05	0.014*	P < 0.001*
vCOP ML (cm/s)	0.67 (0.35)	0.57 (0.22)	1.06 (0.62)	P > 0.05	0.002*	P < 0.001*
Entropy AP	0.15 (0.08)	0.07 (0.03)	0.09 (0.05)	P < 0.001*	P < 0.001*	P = 0.43
Entropy ML	0.14 (0.08)	0.08 (0.04)	0.09 (0.04)	P < 0.001*	0.002*	P > 0.05
Peak power AP (0–0.5 Hz) (cm)	5.36 (3.14)	18.39 (20.30)	19.89 (15.44)	P < 0.001*	0.002*	P > 0.05
Peak power ML (0–0.5 Hz) (cm)	5.83 (5.71)	20.81 (24.20)	21.89 (22.66)	0.009*	0.008*	P > 0.05
Peak power AP (0.5–1 Hz) (cm)	7.53 (6.14)	9.82 (10.05)	20.36 (17.02)	P > 0.05	P < 0.001*	0.003*
Peak power ML (0.5–1 Hz) (cm)	7.91 (7.31)	5.65 (4.06)	16.41 (21.79)	P > 0.05	0.049*	0.009*

BBS–Berg Balance Scale, TUG–Timed Up and Go test, FRT–Functional Reach Test, COP—center of foot pressure, vCOP—velocity of COP, AP—anterio-posterior direction, ML—medio-lateral direction, PD—Parkinson's disease, PD-II—participants with mild PD, HC–healthy control, PD-III—participants with moderate PD, SD—standard deviation

*statistically significant differences between groups (P < 0.05).

### Spatiotemporal COP measures

**Fig 2:**
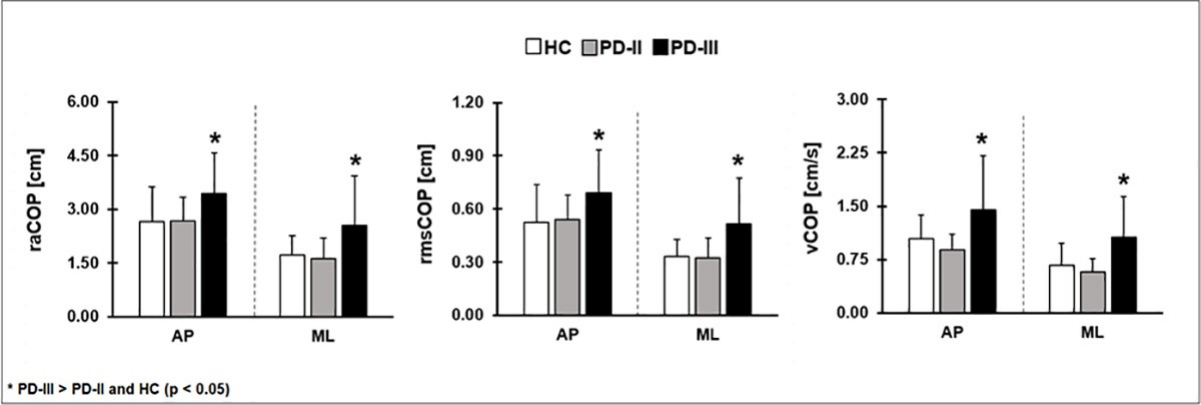
Three different posturographic measurements (range, root mean square and velocity of COP) among HC, PD-II and PD-III. The spatiotemporal measurements were higher in PD-III relative to PD-II and HC; however, they did not differ significantly between HC and PD-II. This finding indicates that the spatiotemporal measurements are insensitive in identifying balance impairments in PD-II relative to HC. AP–anterior-posterior direction, ML–medio-lateral direction, raCOP–range of COP, rmsCOP–root mean square of COP, vCOP–velocity of COP.

We compared the spatiotemporal COP measures among HC, PD-II, and PD-III for the two visual conditions (eyes open vs. eyes closed) for each direction (AP vs. ML). All spatiotemporal COP measures were significantly greater in PD-III relative to both groups PD-II and HC in both anterior-posterior and medio-lateral direction (Fig 2; Table 2). However, there was no significant difference between the two groups (PD-II vs. HC) for any of the spatiotemporal COP measures (P > 0.1; Fig 2, Table 2). The spatiotemporal COP measures were not significantly affected by the visual condition in either direction (P > 0.1). These findings suggest that spatiotemporal measures of the COP could not detect balance impairments in PD-II relative to HC.

### Sample entropy of the COP

**Fig 3:**
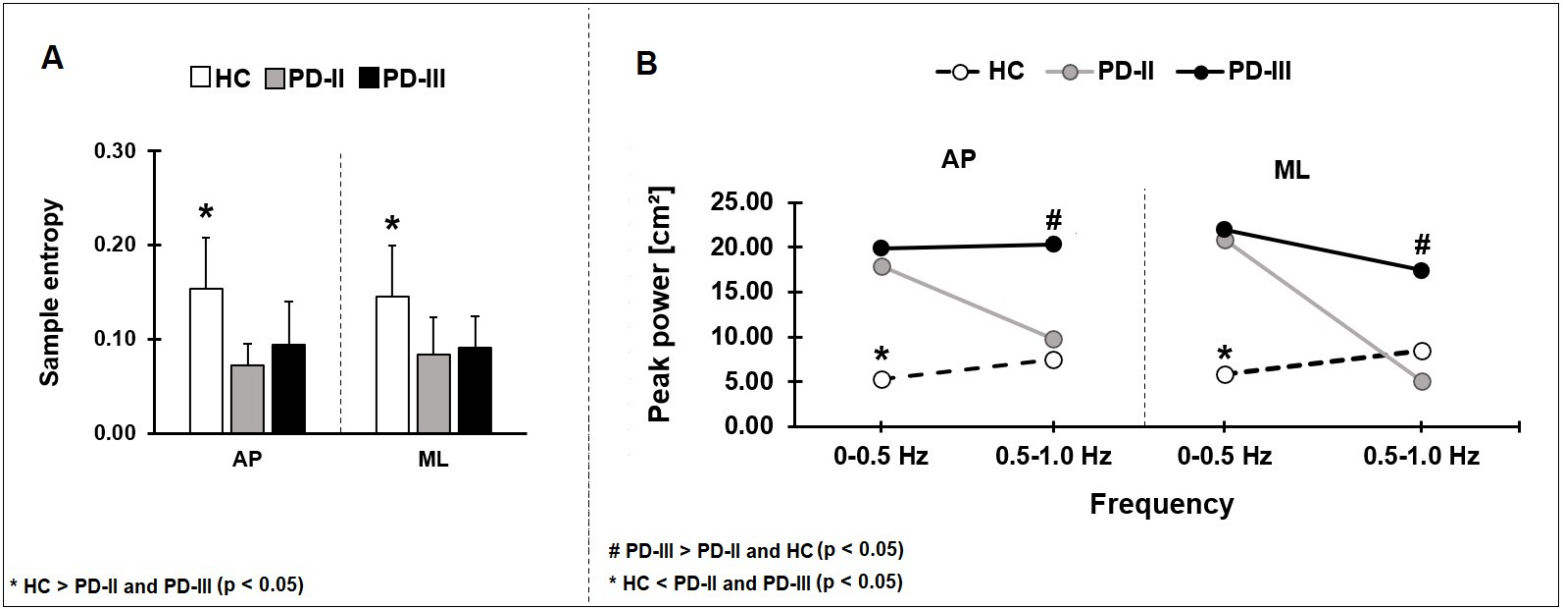
We compared sample entropy (A) and peak power (B) among HC, PD-II and PD-III. Sample entropy was significantly lower in mild and moderate PD participants relative to HC. The peak power was greater at 0–0.5 Hz frequencies bands in both the PD-II and PD-III groups relative to HC and at 0.5–1.0 Hz was greater in PD-III relative to PD-II and HC. AP–anterior-posterior direction, ML–medio-lateral direction.

We compared the sample entropy of COP among HC, PD-II, and PD-III for the two visual conditions (eyes open vs. eyes closed) for each direction (AP vs. ML). Sample entropy was greater in HC relative to both PD groups in both the anterior-posterior and medio-lateral direction (Fig 3A, Table 2). Sample entropy was not significantly affected by the visual condition in either direction (P > 0.1). These findings suggest that although sample entropy detected differences between HC and PD-II, it was insensitive in detecting differences between PD-II and PD-III.

### Power spectral density of the COP

We compared the peak power of COP excursion in two frequency bands (0–0.5 vs. 0.5–1 Hz) among HC, PD-II, and PD-III for the two visual conditions (eyes open vs. eyes closed) for each direction (AP or ML). There was a significant group x frequency band interaction indicating that the groups varied differently for each frequency band (Fig 3B). Specifically, from 0–0.5 Hz, the PD-II and PD-III groups exhibited greater peak power than HC in both the AP and ML direction (Fig 3B; Table 2). The peak power was not significantly different between PD-II and PD-III (P > 0.05; Table 2). In contrast, from 0.5–1 Hz, the PD-III group exhibited greater peak power than the PD-II and HC in both AP and ML directions (Fig 3B; Table 2). The peak power from 0.5–1 Hz was not significantly different between PD-II and HC (P > 0.05; Table 2). The peak power of both frequency bands was not significantly affected by the visual condition in either direction (P > 0.05). These findings suggest that COP oscillations from 0–0.5 Hz detect differences for PD-II relative to HC, whereas COP oscillations from 0.5–1 Hz detect differences between PD-II and PD-III.

## Discussion

Identifying balance deficits in early PD relative to healthy controls (HC) has been difficult and many practitioners have erroneously assumed that postural control issues only manifest in mid to late stage disease. Here, we aimed to identify changes in postural control of PD at early stages of the disease and we compared the findings to HC and moderate PD using clinical measures of balance and posturography. We found that clinical balance tests and standard posturographic measurements were not able to detect balance deficits in early PD, whereas more sophisticated analyses of the COP time series differentiated early PD from HC. Specifically, early PD and moderate PD were characterized by more power from 0–0.5 Hz than HC, whereas only moderate PD exhibited more power from 0.5–1 Hz than early PD and HC. Thus, early PD patients in this small cohort were characterized by distinct frequency structure of the COP from 0–1 Hz. This frequency structure separated the patients from HC and moderate PD.

### Clinical measures of balance

Similar to previous studies [2,25], we observed that clinical measures of balance failed to detect deficits in early PD relative to HC. Specifically, we found that scores on BBS, Tinetti, FRT, and TUG were not significantly different between early PD and HC (see Fig 1). Although, all these tests were reliable tools [26,27], they were only useful for detecting balance deficits in moderate-advanced stages of PD relative to HC [4] and in older adults [28]. For example, moderate PD exhibited lower scores of BBS [29] relative to HC. Also, advanced PD exhibited longer times to perform the TUG test [30,31]. Consistent with previous findings, we also showed that moderate PD had lower scores on BBS, Tinetti, FRT and longer TUG compared with HC and mild PD participants. Thus, the available clinical tests of balance could not distinguish early stage PD from HC.

### Posturography–spatiotemporal measures

In this study, we observed that spatiotemporal measures of COP (raCOP, vCOP, rmsCOP) could not differentiate early PD from HC in both the anterior-posterior and medio-lateral directions. Although at first, this finding seemed to contrast with the previously reported spatiotemporal COP deficits in early PD relative to HC, this discrepancy in findings can be explained by the disease severity of the PD participants. Specifically, Stylianou et al. [32] compared PD patients with HC who had a UPDRS score of 20.5. Thus, the PD participants in this study were comparable to our “moderate” level PD and definitely more advanced than our early PD group who on average had a UPDRS score equal to 10.5. Similarly, Błaszczyk et al. [33] and Rocchi et al. [34] investigated PD participants in all H&Y stages together. The authors concluded that early PD exhibited greater instability in the mediolateral sway however they combined groups. Finally, Chastan et al. [3] showed postural control impairments in early stage PD however these findings are based on a single posturographic measurement (COP sway area) and there were only nine participants. Thus, the findings of the existing studies on early PD were influenced by the level of disease severity and the number of participants.

### Posturography–sample entropy and PSD

Our findings clearly suggest that clinical measures of balance and spatiotemporal measures of COP are both insufficient in detecting changes in postural control for early PD relative to HC (mean UPDRS motor score of 10.5). Thus, in this study we examined the COP time series using more advanced analytical methods. Specifically, we examined the sample entropy and power spectra of the COP, which both provided information about the structure of the COP signal. The sample entropy examine the regularity of the signal and it quantified the signal with a single value. Lower values of sample entropy reflects regular signal, whereas higher values reflects random signal [22]. Greater regularity in the COP signal revealed less effective balance control in children with cerebral palsy [35] and individuals who had a previous stroke [36]. Here, we observed that the sample entropy differed for all PD relative to HC. Specifically, we showed that both early and moderate PD had a sample entropy close to 0.1, whereas HC had a sample entropy close to 0.2. This indicated in our small cohort that the COP time series was more regular in PD than in HC. We therefore would posit that there was some sort of impairment in the postural control system. Nonetheless, this measure failed to differentiate early PD from moderate PD.

Examining the power spectra of the COP time series provided more information about the structure of the signal than the sample entropy. Specifically, the power spectra can provide approximations of the underlying oscillations in the COP and of their respective amplitude. For the COP time series, there is strong evidence that most of the power occurs from 0–1 Hz [12,37–39]. Consistent with the literature, we find that all three groups in this study (early PD, moderate PD, HC) had ~80% of their power within 0–1 Hz. Thus, we examined the oscillations in two distinct bins within 0–1 Hz, as we have done previously [12]. Specifically, we examined the peak power from 0–0.5 Hz and 0.5–1 Hz. We observed that early PD had a characteristic structure for the COP relative to HC and moderate PD. Early PD exhibited more power than HC but comparable power to moderate PD (from 0–0.5 Hz). In contrast, early PD exhibited less power than moderate PD but comparable power to HC (from 0.5–1 Hz). Although there were no previous direct studies to draw from comparing early PD with HC from 0–1 Hz, the importance of these low-frequency oscillations to the postural control has been shown in multiple sclerosis [39]. The authors noticed more power at 0.3–1 Hz in the medio–lateral direction in individuals with multiple sclerosis, what indicates the impaired ability of the somatosensory system in regulation of postural control. Also, there is evidence that different ranges of postural frequency express the different levels of activity of postural subsystems, i.e. frequencies 0–0.3 Hz are associated with visual regulation, frequencies 0.3–1 Hz with vestibular regulation, and frequencies 1–3 Hz with proprioceptive regulation [40,41]. According to these studies it appears that larger sway in each of these frequencies reflects increased activity within the relevant postural subsystem, either due to pathology or to compensatory efforts. In the case of this, our results confirmed postural control changes relevant with Parkinson’s disease. The mild PD participants present more total power at 0–0.5 Hz relative to moderate PD and healthy subjects. However, the more total power present moderate PD group compared to mild stage PD subjects and healthy older people from 0.5–1 Hz. This finding, therefore, highlights the impaired ability of the regulation of postural control even in early affected people with PD, which confirmed our hypothesis about balance impairments in early stage PD.

### Why do low-frequency oscillations in the COP characterize early PD?

Low-frequency oscillations in the COP have been associated with impaired postural control in older adults [12]. The current belief is that oscillations below 0.5 Hz reflect an oscillation that is part of the descending drive to the motor neuron pool, whereas oscillations from 0.5–1 Hz likely reflect visual regulation of the motor output [39,42]. Here, we show that the COP oscillations below 0.5 Hz are exacerbated in early and moderate PD relative to HC with eyes open and eyes closed. Thus, exacerbated oscillations in this frequency band likely reflect a change in the modulation of the descending drive to the motor neuron pool. In contrast, only moderate PD patients exhibited greater power from 0.5–1 Hz. This exacerbation in power could not therefore be due to visual feedback regulation, since the same finding manifests with the eyes open and closed. The finding likely reflects a more widespread exacerbation of low-frequency oscillations in moderate PD relative to early PD. Therefore, exacerbation of low-frequency oscillations likely reflects a loss of control of the descending drive to the motor neuron pool. This loss of power likely originated from degeneration of neurons in brain regions. The exact effect of degenerated brain regions on the modulation of the motor output remains unexplored.

### Limitations

There are two limitations to our study: 1) The current findings are based on a cross-sectional comparison of early PD with moderate PD and HC. 2) PD has at least two subtypes of patients, those who exhibit postural instability and gait difficulties and those who exhibited excessive tremor [43]. Here, we did not differentiate PD participants into the two subtypes, which could have contributed to balance deficits relative to HC. Thus, future studies should incorporate a longitudinal design that will include a larger number of participants drawn from different PD subtypes.

### Conclusion

In summary, the results of the present study revealed that clinical tests and traditional measures of postural sway were unable to differentiate early PD and HC. More advanced measures of COP, such as the sample entropy and power spectral density, detected differences in postural control between HC and early PD. However, only the frequency analysis of the COP time series could differentiate early PD from HC and from moderate PD. Thus, our findings provide evidence that the power spectral analysis of the COP may be a useful tool to detect postural control deficits in early PD. This is important for clinical practice to establish early treatment and proper rehabilitation protocols for PD patients to slow disease progression related to balance impairments.

## Supporting information

S1 Data (XLSX)
